# Ipsilateral Motor Pathways after Stroke: Implications for Non-Invasive Brain Stimulation

**DOI:** 10.3389/fnhum.2013.00184

**Published:** 2013-05-08

**Authors:** Lynley V. Bradnam, Cathy M. Stinear, Winston D. Byblow

**Affiliations:** ^1^Brain Research Laboratory, Centre for Neuroscience, School of Medicine, Flinders UniversityAdelaide, SA, Australia; ^2^Effectiveness of Therapy Group, Centre for Clinical Change and Healthcare Research, School of Medicine, Flinders UniversityAdelaide, SA, Australia; ^3^Clinical Neuroscience Laboratory, Department of Medicine, The University of AucklandAuckland, New Zealand; ^4^Centre for Brain Research, The University of AucklandAuckland, New Zealand; ^5^Movement Neuroscience Laboratory, The University of AucklandAuckland, New Zealand

**Keywords:** stroke, rehabilitation, upper limb, propriospinal, transcranial direct current stimulation

## Abstract

In humans the two cerebral hemispheres have essential roles in controlling the upper limb. The purpose of this article is to draw attention to the potential importance of ipsilateral descending pathways for functional recovery after stroke, and the use of non-invasive brain stimulation (NBS) protocols of the contralesional primary motor cortex (M1). Conventionally NBS is used to suppress contralesional M1, and to attenuate transcallosal inhibition onto the ipsilesional M1. There has been little consideration of the fact that contralesional M1 suppression may also reduce excitability of ipsilateral descending pathways that may be important for paretic upper limb control for some patients. One such ipsilateral pathway is the cortico-reticulo-propriospinal pathway (CRPP). In this review we outline a neurophysiological model to explain how contralesional M1 may gain control of the paretic arm via the CRPP. We conclude that the relative importance of the CRPP for motor control in individual patients must be considered before using NBS to suppress contralesional M1. Neurophysiological, neuroimaging, and clinical assessments can assist this decision making and facilitate the translation of NBS into the clinical setting.

## Introduction

Reaching forward with the arm to manipulate objects with the hand is a quintessential function for higher order primates. Upper limb movements involve a fine balance between proximal stability and distal dexterity, presenting a unique motor control challenge to the central nervous system. There is a growing body of evidence that skilled upper limb function is under the control of both contralateral (cM1) and ipsilateral (iM1) motor cortices (Chen et al., 1997; Gerloff et al., 1998; Muellbacher et al., 2000; Hummel et al., 2003; Sohn et al., 2003; Verstynen et al., 2005; Davare et al., 2007; Duque et al., 2008; Perez and Cohen, 2008, 2009; Lee et al., 2010). Exactly how iM1 contributes to ipsilateral upper limb control is unclear, and is likely to involve both interhemispheric and descending projections. Neurophysiological studies have shown that iM1 assists cM1 to shape motor output by modulating the degree of transcallosal inhibition between homologous muscle representations in the two hemispheres (Sohn et al., 2003; Davare et al., 2007; Perez and Cohen, 2008). The potential importance of descending pathways from iM1 to spinal cord for upper limb control has largely been ignored. In this paper we present a novel hypothesis to account for how iM1 contributes to skilled upper limb motor control. We propose that the pathway involves a robust ipsilateral projection called the cortico-reticulo-propriospinal pathway (CRPP), based on findings in the cat and non-human primate (Illert et al., 1981; Alstermark et al., 1984; Isa et al., 2006). The CRPP descends from iM1 via the reticulospinal tract and terminates on propriospinal neurons (PNs) located at C3/4 in the spinal cord (Alstermark et al., 2007). PNs project to alpha motoneurons (αMNs) innervating muscles involved in specific tasks so movements can be rapidly generated and modified as necessary (Pierrot-Deseilligny and Burke, 2005). Our hypothesis is that neural inputs from the CRPP are integrated by PNs with those from the disynaptic (indirect) portion of the contralateral corticospinal tract. As a result, descending inputs from both hemispheres shape the final motor command reaching αMNs innervating upper limb musculature for optimal movement control.

Up-regulation of contralesional motor cortex excitability and the CRPP pathway may be important for paretic arm function after stroke (Turton et al., 1996; Netz et al., 1997; Alagona et al., 2001; Lewis et al., 2004; Misawa et al., 2008), particularly in poorly recovered patients (Turton et al., 1996; Netz et al., 1997; Gerloff et al., 1998; Caramia et al., 2000; Trompetto et al., 2000; Lewis and Perreault, 2007; Misawa et al., 2008). The degree of reorganization toward contralesional hemisphere control may depend on the residual integrity of white matter tracts from the ipsilesional hemisphere (Ward et al., 2006, 2007; Stinear et al., 2008; Grefkes and Fink, 2011). The neurophysiological model proposed here explains how increased excitability of the CRPP disrupts the normal cM1-iM1 balance of descending inputs reaching C3/4 PNs. In patients with a relatively intact ipsilesional corticospinal tract, up-regulation of the CRPP pathway would interfere with descending commands to PNs from the ipsilesional cortex. The model also accounts for why the CRPP is integral to residual function when the ipsilesional corticospinal tract is severely compromised. In these patients the CRPP may be the only intact descending pathway from cortex to spinal cord, and therefore of particular importance for their motor recovery.

Finally, a contribution by contralesional M1 to upper limb motor control via the CRPP has implications for NBS protocols aimed at improving rehabilitation of the paretic upper limb after stroke. The proposed model shows that contralesional M1 suppression after NBS may affect stroke patients differently depending on the severity of damage to the ipsilesional corticospinal tract and the degree of up-regulation of the contralesional CRPP. Studies that have included more severely impaired patients seem to indicate paretic upper limb motor performance is degraded by contralesional M1 NBS (Ackerley et al., 2010; Theilig et al., 2011; Bradnam et al., 2012). We propose that NBS protocols that aim to suppress contralesional M1 may be contraindicated for some patients. We argue that NBS is not a “one size fits all” solution for recovery after stroke, but that it can be tailored to individual patients based on neurophysiological and clinical biomarkers that are relative easy to obtain (Stinear et al., 2007, 2012; Jang et al., 2010; Kwon et al., 2011; Riley et al., 2011).

## A Neurophysiological Model for Ipsilateral Upper Limb Control

**Figure 1:**
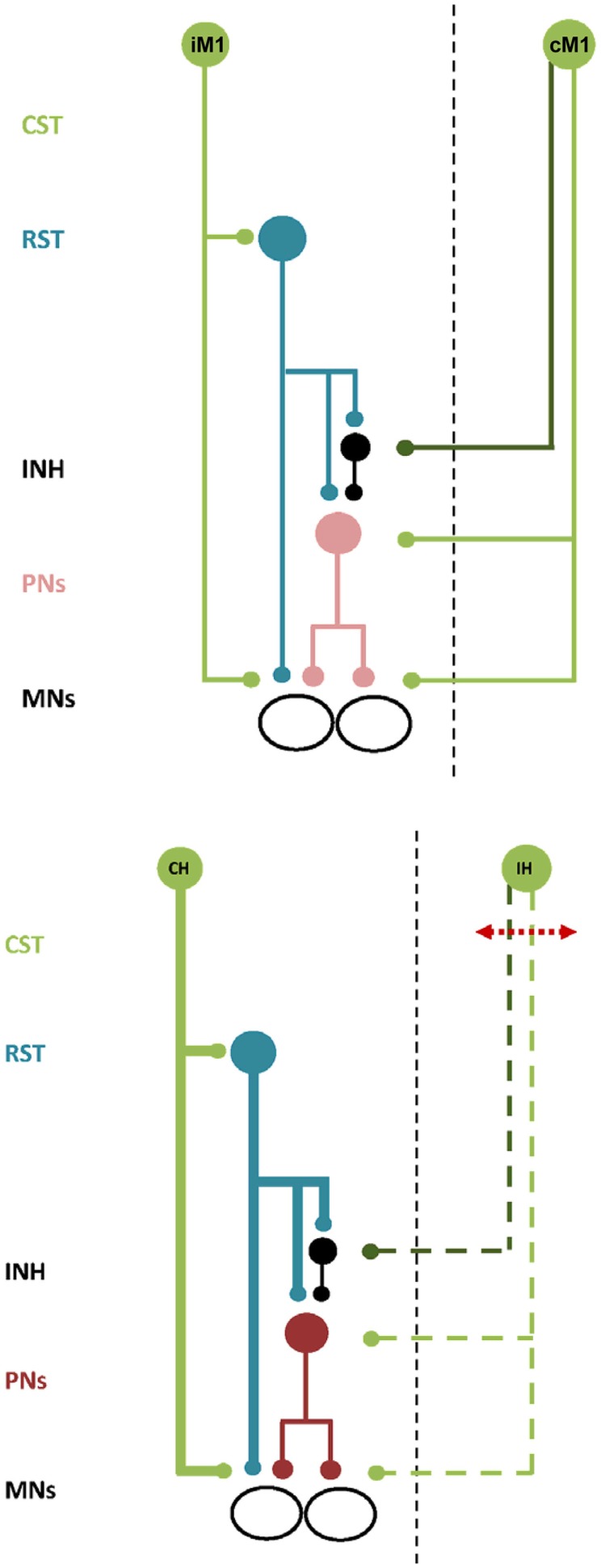
**Schematic illustration of bilateral neural control and ipsilateral neural control after stroke. Top**. Descending commands to presumed PNs and inhibitory interneurons via the ipsilateral cortico-reticulo-propriospinal tract (CRPP) and the contralateral corticospinal tract provides balanced input to αMNs in the spinal cord. All projections are facilitatory except for the inhibitory interneuron, shown in black. CST, corticospinal tract. RST, reticulospinal tract. INH, inhibitory interneuron. PNs, propriospinal neurons. MNs, alpha motoneurons. IM1 and CM1, ipsilateral and contralateral primary motor cortex. **Bottom**. There is compensatory up-regulation of the contralesional hemisphere resulting in greater excitability of the ipsilateral CRPP (bold blue lines) and disruption to facilitatory and inhibitory descending inputs from the ipsilesional hemisphere (dashed green lines). PNs are facilitated resulting in impaired motor control of the paretic upper limb. All projections are facilitatory except for the inhibitory interneuron, shown in black. Abbreviations as above except for CH and IH, indicating the contralesional and ipsilesional hemisphere respectively.

Many tasks performed with one hand are challenging and require precise co-contraction of multiple muscles across the upper limb. Skilled and complex unimanual movements are accompanied by an increase in iM1 excitability (Hummel et al., 2003; Verstynen et al., 2005; Morishita et al., 2011; Uehara et al., 2011), potentially by modulation of transcallosal projections (Perez and Cohen, 2008; Morishita et al., 2011; Uehara et al., 2011)_ENREF_8 or by increased transmission through ipsilateral projections to spinal αMNs (Gerloff et al., 1998). Using Transcranial Magnetic Stimulation (TMS), iM1 has been found to contribute to sequential movement timing and selective activation of proximal and distal muscles in healthy adults (Gerloff et al., 1998; Carey et al., 2006; Davare et al., 2007; Duque et al., 2008; Bradnam et al., 2010; McCambridge et al., 2011). It appears that muscle coordination for precise and skilled unimanual tasks requires activation of both cortical hemispheres. The neurophysiological model of bilateral motor control from cM1 and iM1 is illustrated schematically in Figure 1. Both cortical hemispheres have direct and indirect projections to αMNs in the spinal cord (Kuypers, 1964; Brinkman and Kuypers, 1973). From cM1, the corticospinal tract descends in the internal capsule to the brainstem and onto the spinal cord where the monosynaptic (direct) portion terminates onto αMNs in the ventral horn (Fries et al., 1993). The disynaptic (indirect) portion of the tract originates from distinct populations of neurons in cM1 and descends alongside the rubrospinal and tectospinal tracts to converge onto cervical PNs (Alstermark et al., 2007; Lemon, 2008). Ipsilateral descending motor control is also mediated by direct and indirect pathways. From iM1, approximately 10–15% of the corticospinal fibers are uncrossed and project directly to αMNs in the ipsilateral spinal cord. The indirect ipsilateral descending tract is the CRPP. The proposed anatomical pathway is as follows. After traversing the internal capsule separately, descending projections from the ipsilateral premotor cortex and iM1 terminate on reticular neurons in the brainstem that give rise to the reticulospinal descending tracts (Andrews et al., 1973; Catsman-Berrevoets and Kuypers, 1976). The reticulospinal projections descend in the spinal cord as inhibitory (lateral) and excitatory (medial) tracts, terminating on medial motor nuclei in the spinal cord to innervate axial and proximal limb αMNs (Kuypers, 1964; Brinkman and Kuypers, 1973). In the non-human primate, reticulospinal tracts provide bilateral innervation to muscles of the proximal upper limb (Davidson and Buford, 2004, 2006; Davidson et al., 2007).

In the cat and non-human primate reticulospinal projections converge onto PNs (Brinkman and Kuypers, 1973; Illert et al., 1977, 1981; Alstermark et al., 1984; Boudrias et al., 2010) in addition to αMNs. PNs project to αMNs innervating agonist proximal and distal muscles across the upper limb and to Ia inhibitory interneurons controlling αMNs of antagonist muscles (Tantisira et al., 1996). Descending and ascending inhibitory control over PNs facilitates fine-tuning and precise shaping of motor commands prior to their execution by αMNs. Behavioral studies in the cat show that partially transecting the spinal cord at C3-4 induces dysmetria and discoordination of the forelimb (Alstermark et al., 2007). In humans, the cervical propriospinal system is a candidate neural network underpinning muscle synergies for upper limb reaching (Pierrot-Deseilligny and Burke, 2005). Task-specific use of the hand disinhibits PNs to activate proximal wrist and shoulder muscle synergies important for limb stabilization (Iglesias et al., 2007; Roberts et al., 2008; Giboin et al., 2012). According to our hypothesis, descending commands from the CRPP are integrated with those from cM1 via the disynaptic (indirect) corticospinal tract within presumed C3/4 PNs. The balance of descending facilitation and inhibition from the two hemispheres dictates overall spinal PN excitability and subsequent activation of αMNs. There is evidence in humans for an ipsilateral pathway from iM1 to spinal PNs in support of our model. c-tDCS applied to iM1 suppressed both facilitatory and inhibitory projections to ipsilateral PNs in the spinal cord of healthy adults (Bradnam et al., 2011), although involvement of the reticulospinal tract cannot be confirmed from this experiment. It also appears possible to indirectly modulate both upper and lower limb PN circuits using NBS over M1 (Bradnam et al., 2011; Roche et al., 2011, 2012). ENREF_31 Future experiments may further elucidate how PNs integrate bilateral descending commands from motor cortex and the role of the cervical propriospinal system in complex upper limb tasks in humans.

Inhibitory projections to PNs may originate in premotor cortex rather than M1 (see Figure 1, top panel). This is important as descending tracts from premotor cortex are separated from those of M1 within the internal capsule. White matter tracts from premotor cortex descend in the genu and anterior portion of the posterior limb of the internal capsule, while those from M1 descend in the posterior limb itself (Fries et al., 1993). Therefore, the location of a stroke lesion in the internal capsule would have a significant impact on the residual ability of ipsilesional M1 to provide inhibitory modulation of PNs and αMNs.

## Neurophysiological Model of Ipsilateral Upper Limb Control after Stroke

After stroke there can be an increase in motor cortex excitability in the contralesional hemisphere and reduced excitability of the ipsilesional hemisphere. This view of interhemispheric imbalance of the motor system after stroke occurs via transcallosal inhibitory pathways between the two hemispheres (Shimizu et al., 2002; Murase et al., 2004; Ward et al., 2006, 2007; Grefkes et al., 2008; Ameli et al., 2009; Grefkes and Fink, 2011). Within the first 2 weeks after stroke contralesional hemispheric excitability can increase in patients with severe deficits (Rehme et al., 2011). The degree of interhemispheric imbalance in excitability and motor impairment may be negatively correlated with the residual integrity of the ipsilesional corticospinal tract after the stroke (Grefkes and Fink, 2011). This is supported by evidence that contralesional hemisphere activity is enhanced in patients with more extensive ipsilesional corticospinal tract disruption and greater upper limb impairment (Johansen-Berg et al., 2002; Ward et al., 2003, 2006, 2007; Lotze et al., 2006; Stinear et al., 2008). In well-recovered patients contralesional hemisphere excitability decreases over time (Stinear et al., 2008). In severely affected patients, contralesional hemisphere excitability gradually increases as motor function recovers, indicating reorganization toward the contralesional hemisphere (Stinear et al., 2008). This increase in contralesional M1 excitability is also marked by facilitation of ipsilateral descending projections to paretic upper limb muscles (Turton et al., 1996; Netz et al., 1997; Caramia et al., 2000; Alagona et al., 2001; Lewis and Perreault, 2007).

Cortical reorganization following spinal cord transection consists of an early phase of bilateral M1 up-regulation, followed later by increased contralesional M1 and bilateral premotor excitability (Nishimura et al., 2007; Nishimura and Isa, 2009). Hand dexterity can be restored by intensive rehabilitation following transection of the contralateral corticospinal tract in brain or cervical cord but is accompanied by abnormal co-activation of distal and proximal muscles (Nishimura et al., 2009), in particular forearm and hand flexors (Zaaimi et al., 2012). Furthermore, these studies have demonstrated there is only a weak contribution of ipsilateral M1 to residual motor control of the paretic upper limb (Schmidlin et al., 2004; Zaaimi et al., 2012), indicating intact subcortical descending pathways are responsible for the observed motor recovery (Nardone et al., 2013). Together these studies support an emerging concept of reorganization via ipsilateral reticulospinal and propriospinal systems following disruption to contralateral corticospinal tract in line with our hypothesis.

A model for how the ipsilateral CRPP may influence upper limb recovery after stroke is illustrated schematically in Figure 1, bottom panel. The model demonstrates that the balance of cM1-iM1 descending inputs reaching C3/4 PNs can be affected by the stroke lesion. Descending motor control by the ipsilesional hemisphere is reduced with disruption to the corticospinal tract. This is associated with an increase in excitability of contralesional M1 and descending output along the ipsilateral CRPP, leading to a worsening of upper limb impairment. Why might this occur? Ipsilesional white matter damage confined to the – posterior limb of the internal capsule may spare the descending inhibitory projections originating in the premotor cortex that traverse the genu of the internal capsule and the ventral portion of the posterior internal capsule (Fries et al., 1993). A moderate increase in presumed PN excitability via the CRPP could be balanced by the residual inhibitory control over PNs from the ipsilesional hemisphere. In contrast, extensive damage to the ipsilesional descending pathways would compromise both excitatory *and* inhibitory projections to presumed PNs. With greater tract damage there would be little remaining ipsilesional inhibitory control to counterbalance the significant facilitation of presumed PNs via the contralesional CRPP. It is also possible that small lesions may affect the CRPP in isolation and give rise to abnormal synergistic control through altered input to PNs and inhibitory interneurons. This model of ipsilateral CRPP up-regulation is supported by experiments showing that excitability of circuits mediated by presumed PNs is increased after stroke (Mazevet et al., 2003; Stinear and Byblow, 2004), suggesting up-regulation of these indirect descending pathways when the corticospinal tract is compromised.

The model predicts that *contralesional* CRPP influences upper limb function negatively to positively along a continuum that depends on *ipsilesional* white matter tract integrity. In patients with minimal damage to the corticospinal tract, the contralesional CRPP may interfere with residual descending inputs from ipsilesional hemisphere, at the level of the spinal cord. Greater descending drive through the ipsilateral reticulospinal tract compared to contralateral corticospinal tract may result in aberrant recruitment of PNs and inhibitory interneurons. In the presence of extensive damage, the contralesional CRPP may provide the only descending cortical commands to reach the spinal cord. This pathway can only partially compensate for the loss of excitatory and inhibitory descending control from ipsilesional M1. The loss of tonic inhibition over αMNs, and aberrant control of PNs, might explain the emergence of abnormal muscle synergies and spasticity in the paretic upper limb in more severely impaired patients after stroke. This model highlights the importance of the contralesional CRPP pathway for residual control over the paretic upper limb, which may have relevance for the use of NBS protocols that aim to suppress the contralesional M1.

## Implications for Non-Invasive Brain Stimulation

**Figure 2:**
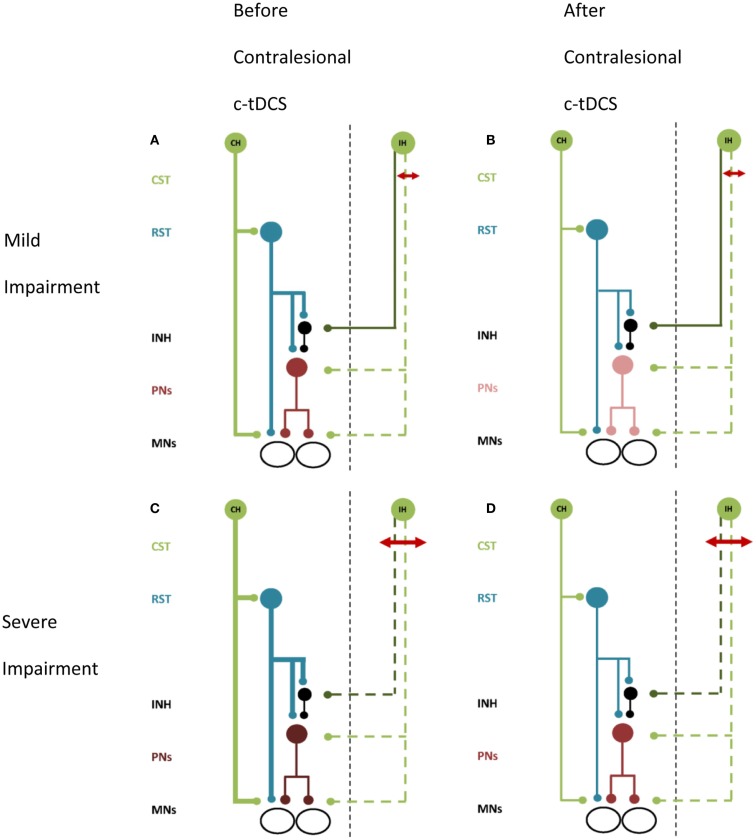
**A schematic of proposed effects of contralesional M1 c-tDCS**. Line thickness indicates relative excitability, thicker lines represent greater excitability. Red arrow indicates the stroke lesion. Note **(A,B)** lesion affects excitatory projections (i.e., the posterior limb of the internal capsule), **(C,D)** lesion affects both excitatory and inhibitory projections (i.e., most of internal capsule). **(A)** Patients with mild upper limb impairment. There is up-regulation of the CRPP from contralesional M1 and residual ipsilesional inputs to PNs and inhibitory interneurons. **(B)** Mildly impaired patients after contralesional M1 c-tDCS. Contralesional M1 and the CRPP are suppressed removing interference with ipsilesional inputs to PNs. Inhibition over PNs is restored and paretic upper limb motor control is improved. **(C)** Patients with moderate to severe upper limb impairment. There is greater up-regulation of contralesional M1 and little or no descending input to PNs and inhibitory interneurons from ipsilesional M1. **(D)** Severely impaired patients after contralesional M1 c-tDCS. Contralesional M1 and CRPP excitability is reduced removing PN facilitation. Motor control is worsened. All projections are facilitatory except for the inhibitory interneuron, shown in black. Abbreviations as for Figure 1.

Non-invasive brain stimulation may be a useful adjuvant to re-balance motor cortex excitability after stroke, with the aim of improving motor function of the paretic upper limb during recovery (Hummel et al., 2008). Using NBS to directly facilitate ipsilesional M1 can improve paretic hand function by increasing excitability of descending projections to αMNs (Fregni et al., 2005; Mansur et al., 2005; Takeuchi et al., 2005; Boggio et al., 2007; Nowak et al., 2008; Ackerley et al., 2010; Kim et al., 2010; Stagg et al., 2012). Conversely, NBS to suppress excitability of contralesional M1 may restore the balance of hemispheric excitability and also enhance corticomotor drive from ipsilesional M1 to the paretic upper limb (Takeuchi et al., 2005; Suppa et al., 2008; Grefkes et al., 2010). Improvements in hand function have been reported in studies using NBS to suppress contralesional M1 (Fregni et al., 2005; Boggio et al., 2007; Dafotakis et al., 2008; Nowak et al., 2008; Grefkes et al., 2010; Kim et al., 2010), however many studies have examined relatively well-recovered patients. In patients with more severe impairment suppression of the contralesional M1 has been equivocal for improving upper limb function (Ackerley et al., 2010; Theilig et al., 2011; Bradnam et al., 2012; Talelli et al., 2012). The prevailing view of interhemispheric imbalance of motor cortex excitability after stroke, and use of NBS to suppress contralesional M1 to redress imbalance, has not considered effects on output pathways other than transcallosal projections. Our model proposes that suppression of contralesional M1 can reduce excitability of the ipsilateral CRPP (Bradnam et al., 2011), and therefore the use of NBS for suppressing contralesional M1 should be considered carefully. The effects of suppressing contralesional M1 may be quite different for mildly impaired (Figures 2A,B) versus moderate to severely impaired (Figures 2C,D) patients, as illustrated schematically in Figure 2. In a study of patients with upper limb weakness after subcortical stroke, c-tDCS improved paretic proximal upper limb motor control in mildly impaired patients but degraded control in moderate to severely impaired patients (Bradnam et al., 2012). The residual structural integrity of the ipsilesional corticospinal tract was compromised in patients for whom suppression of the contralesional M1 was detrimental. It is conceivable that these patients rely more strongly on compensatory up-regulation of the contralesional M1 and CRPP. Suppression of contralesional M1 may reduce residual descending drive to αMNs, further degrading paretic upper limb motor control. Conversely, for mildly impaired patients, suppressing contralesional M1 may reduce interhemispheric inhibition of ipsilesional M1 leading to improved function of the paretic upper limb as described previously (Fregni et al., 2005; Boggio et al., 2007; Dafotakis et al., 2008; Nowak et al., 2008; Grefkes et al., 2010; Kim et al., 2010). Contralesional M1 suppression also decreases excitability of the contralesional CRPP and could therefore reduce interference between descending inputs to the cord from intact ipsilesional descending pathways and the contralesional CRPP.

This idea has several practical implications. First, measuring the effects of NBS on corticomotor excitability in contralateral distal hand muscles does not completely capture all the effects on descending pathways that reach spinal motoneurons. Second, suppression of contralesional M1 may be contraindicated in more severely affected stroke patients (Bradnam et al., 2012). Third, the proposed CRPP model supports the emerging view that NBS neuromodulation should be tailored individually for patients after stroke based on impairment level (at the chronic stage) (Cramer, 2010; Stinear, 2010; Bradnam et al., 2012) and perhaps, based on the residual capacity of their descending motor pathways (at the sub-acute stage). Further research examining the full range of neurophysiological effects induced by NBS seems warranted. For example, it may be advantageous to use NBS to either facilitate or attenuate contralesional hemisphere excitability based on the degree of damage to the ipsilesional descending pathways. This remains to be determined.

How might individualization of NBS be implemented to improve post-stroke rehabilitation? The presence or absence of motor evoked potentials from TMS and measures of structural integrity derived from diffusion-weighted imaging (DWI) provide objective information about corticospinal tract integrity. TMS and DWI can be used in combination with clinical measures to predict the potential for recovery of function with rehabilitation (Stinear et al., 2007, 2012; Jang et al., 2010; Kwon et al., 2011; Riley et al., 2011). TMS and DWI could also determine which patients are suitable for suppressive NBS to contralesional M1. A recent study indicated that patients with moderate damage to the internal capsule did not have a positive response to contralesional M1 c-tDCS (Bradnam et al., 2012). Patients in whom motor control was improved by NBS were characterized by the presence of responses to TMS from stimulation of ipsilesional M1, and low impairment levels or no upper limb spasticity as assessed by clinical scales. These relatively simple measures could be used to help decide whether a given NBS protocol is appropriate for individual patients.

## Conclusion

It is becoming increasingly clear that skilled function of the upper limb relies on the balance of excitability of cM1 and iM1 in healthy adults. In this article we propose a neurophysiological model of ipsilateral neural control of the proximal upper limb via the CRPP. The model highlights the importance of understanding how the degree of compensatory activity in the contralesional hemisphere after stroke contributes to paretic upper limb function across a range of impairment levels. It is proposed that NBS protocols are not “one size fits all” and should be carefully selected based on individual patient characteristics. Research has targeted the development of structural and functional biomarkers that can be combined with clinical tests to allow NBS to be individually prescribed as an adjuvant to therapy after stroke. These methods of patient stratification based on objective measures of impairment should be used to determine the optimum intervention in future studies of NBS in stroke. Individualization may increase effectiveness of NBS in clinical trials and expedite translation from research laboratory to the clinical setting.

## Conflict of Interest Statement

The authors declare that the research was conducted in the absence of any commercial or financial relationships that could be construed as a potential conflict of interest.
